# Fine Mapping and Whole-Genome Resequencing Identify the Seed Coat Color Gene in *Brassica rapa*

**DOI:** 10.1371/journal.pone.0166464

**Published:** 2016-11-09

**Authors:** Yanhua Wang, Lu Xiao, Shaomin Guo, Fengyun An, Dezhi Du

**Affiliations:** Key Laboratory of Spring Rapeseed Genetic Improvement, The Qinghai Research Branch of the National Rapeseed Genetic Improvement Center, Academy of Agricultural and Forestry Sciences, Qinghai University, Xining, Qinghai, China; Huazhong University of Science and Technology, CHINA

## Abstract

A yellow seed coat is a desirable agronomic trait in the seeds of oilseed-type *Brassica* crops. In this study, we identified a candidate gene for seed coat color in Dahuang, a landrace of *Brassica rapa*. A previous study of Dahuang mapped the seed coat color gene *Brsc1* to a 2.8-Mb interval on chromosome A9 of *B*. *rapa*. In the present study, the density of the linkage map for *Brsc1* was increased by adding simple sequence repeat (SSR) markers, and the candidate region for *Brsc1* was narrowed to 1.04 Mb. In addition, whole-genome resequencing with bulked segregant analysis (BSA) was conducted to identify candidate intervals for *Brsc1*. A genome-wide comparison of SNP profiles was performed between yellow-seeded and brown-seeded bulk samples. SNP index analyses identified a major candidate interval on chromosome A9 (A09:18,255,838–18,934,000, 678 kb) containing a long overlap with the target region recovered from the fine mapping results. According to gene annotation, *Bra028067* (*BrTT1*) is an important candidate gene for *Brsc1* in the overlapping region. Quantitative reverse transcription (qRT)-PCR revealed that *BrTT1* mainly functions in the seed. Point mutations and small deletions in *BrTT1* were found between yellow- and brown-seeded Dahuang plants. Collectively, the expression and sequence analysis results provide preliminary evidence that *BrTT1* is a candidate gene for the seed coat color trait in Dahuang.

## Introduction

Yellow seed coat is typically a desirable trait in rapeseed breeding. Compared to dark-seeded varieties, yellow rapeseeds have a variety of desirable agronomic traits, including a thinner seed coat, lower husk proportion, lower crude oil pigment content and 5–7% higher oil content [1–3]. *Brassica rapa* L. (2n = 20, AA) is a major vegetable and oilseed crop in China, India and Bangladesh and is a parent species of *Brassica napus* L. (2n = 38, AACC) [4,5]. Yellow-seeded varieties of oilseed-type *Brassica* crops (such as ‘Dahuang’ and ‘Yellow Sarson’ in *B*. *rapa*) have many advantages over their dark-seeded (black or brown) counterparts [6–8]. Therefore, the analysis of genes that induce yellow-seeded germplasm in *B*. *rapa* is important for breeding and research. However, the molecular mechanism of seed coat color formation in *B*. *rapa* is not well understood. One or two genes are thought to be involved in the control of seed coat color in *B*. *rapa* [9–11]. A single gene was reported to control the difference between the brown-seeded Indian ‘Toria’ lines and the yellow-seeded ‘Yellow Sarson’ lines [7], and similar results were found for other yellow-seeded varieties of *B*. *rapa* [8,12]. Genetic maps constructed using various molecular markers and large mapping populations have been used to locate yellow-seed genes in *B*. *rapa*. Several major genes or QTLs for seed coat color have been found in multiple yellow-seeded lines of *B*. *rapa* [13–16].

Bulked segregant analysis (BSA) provides a simple and effective alternative strategy for identifying molecular markers that are linked to target genes by genotyping only a pair of bulked DNA samples from two sets of individuals with distinct or extremely opposite phenotypes [17]. With the rapid development of next-generation sequencing (NGS) technologies, new strategies have been proposed to take advantage of the power of BSA and high-throughput genotyping; these strategies are useful for identifying QTLs or candidate genes. Lee et al. [18] increased the density of a previous genetic map in *Brassica oleracea* by adding new derived cleaved amplified polymorphic sequences (dCAPs) and SNP markers developed by the whole-genome resequencing of two cabbage parental lines and genome-wide SNP identification. One major QTL and three minor QTLs for black rot were identified using the new genetic map [18]. Lu et al. [19] identified the candidate gene *Ef1*.*1* for early flowering in cucumber by BSA and whole-genome resequencing. Abe et al. [20] and Takagi et al. [21] identified the major loci for important agronomic and resistance-related traits by the whole-genome resequencing of pooled DNA from segregating populations of rice.

Dahuang, a yellow-seeded landrace of *B*. *rapa*, originates from Huangyuan County in Qinghai Province. Dahuang not only has the desirable traits of most yellow-seeded cultispecies of *B*. *rapa* but also shows self-compatibility and a higher 1,000-grain weight (6–7 g vs. 5 g) [6]. Therefore, the study of seed coat color in Dahuang is very important for *B*. *rapa* breeding programs. Based on previous studies that mapped the seed coat gene *Brsc1* in Dahuang [6], researchers have developed new molecular markers [22,23]. In this study, we added additional simple sequence repeat (SSR) markers with the aim of delimiting the *Brsc1* gene within a shorter interval. Fine mapping, whole-genome resequencing and quantitative reverse transcription (qRT)-PCR were performed to screen strong candidate genes for *Brsc1*.

## Materials and Methods

### Materials

Two B. *rapa* lines, Dahuang (bright yellow seeds) and 09A-126 (brown seeds), were self-pollinated for more than 8 generations, thus yielding stable seed coat coloration. Using 09A-126 as the donor parent and Dahuang as the recurrent parent, a near-isogenic line known as the BC4 population was constructed after 4 consecutive selective backcrosses. These offspring were then divided into yellow- and brown-seeded groups. Several yellow- and brown-seeded individuals from the BC4 population were chosen to obtain homozygous offspring by selfing for two generations. The homozygous yellow- and brown-seeded offspring were termed BC4F2-Y and BC4F2-B plants, respectively. The field trial was performed in the test field of Qinghai University and no specific permissions were required for these activities. The field studies did not involve endangered or protected species.

### Fine mapping of the yellow-seed gene Brsc1

The BC4 population, which contained 1,739 individuals, was used to finely map the yellow-seed gene *Brsc1* in Dahuang. Total DNA was extracted from leaves using the CTAB method [24]. The seed coat colors of BC4 population individuals were determined after the seed matured. Two yellow-seeded gene pools were constructed using 12 randomly selected yellow-seeded plant DNA extracts from BC4, and 2 brown-seeded gene pools were similarly constructed. These 4 gene pools were used to screen molecular markers in subsequent steps. New SSR markers were developed according to the reference genome sequence near *Brsc1* in the BRAD database (http://brassicadb.org/brad/). Existing SSR markers around the interval where *Brsc1* is located were also downloaded from BRAD. Markers with tight linkage to *Brsc1* were then used to screen recombinant individuals in the BC4 population to calculate their genetic distances using the Kosambi function [25]. The addition of these new markers increased the density of the genetic map near *Brsc1*. A physical map of *Brsc1* was also constructed by synteny analysis between molecular markers and the genome of *B*. *rapa* in the BRAD database. With these new markers, we expected to narrow the known interval where *Brsc1* is located on the chromosome, thereby allowing the identification of candidate genes for seed color from genomic data.

### Localization of Brsc1 by whole-genome resequencing

Whole-genome resequencing was performed on the parents and on 2 bulked samples (bulks) that were constructed by separately mixing the DNA of 20 yellow-seeded plants and 20 brown-seeded plants from the BC4 population. Sequencing was performed as follows. First, DNA was isolated using the CTAB method as described above. Next, DNA-seq libraries were generated using the TruSeq Nano DNA HT Sample Preparation Kit (Illumina, USA). Briefly, DNA samples were fragmented by sonication to 350 bp, and the DNA fragments were blunt end-repaired, A-tailed, and ligated with full-length adapters for PCR amplification. Finally, the PCR products were purified (AMPure XP) and sequenced on an Illumina HiSeq 2500. The reference genome of *Brassica rapa* was downloaded from the Ensembl Genomes database (//ftp.ensemblgenomes.org/pub/plants/release-24/fasta/brassica_rapa/dna/Brassica_rapa.IVFCAASv1.24.dna_sm.toplevel.fa.gz). After quality control, sequence data from the 4 samples were aligned to the reference genome using the Burrows-Wheeler Aligner (parameters: mem -t 4 -k 32 –M), and repetitive sequences were removed using SAMTOOLS (parameter: rmdup) [26]. SNP calling was performed with the UnifiedGenotyper in GATK [27]. The homozygous SNPs between the 2 parents were extracted, and 09A-126 was chosen as the reference. Read depth information for the above homozygous SNPs in the offspring pools was used to calculate the SNP index, which is derived from the ratio of the number of different alleles to the total number of reads for each SNP. Using a sliding window (with a window size of 1 Mb and a step size of 1 kb), the chromosomal distribution of SNP indices for the 2 offspring pools was calculated. Differences between the SNP indices of the 2 pools were calculated as the ΔSNP index. Candidate regions for *Brsc1* were extracted from the chromosomes with 95% confidence intervals [21]. Resequencing and candidate interval determination were performed by Novogene (Beijing, China). Based on the location of *Brsc1*, candidate genes within the candidate interval were selected for annotation according to linear comparative analysis in BRAD and homologous gene analysis in *A*. *thaliana* (TAIR) database (http://www.arabidopsis.org/). The gene that was most closely related to seed coat color was chosen for further analysis.

### Expression profiling analysis of the candidate gene

Seeds from BC4F2-Y and BC4F2-B parents at different developmental stages (7, 14, 21, 28, 35, 42, and 49 days after pollination, DAP) were chosen for total RNA extraction using the EasySpin Plant RNA Kit (Biomed, Beijing, China). RNA was also extracted from other plant tissues, such as roots, stems, leaves, flowers and buds, at 7 DAP. RNA (1 μg) was used to synthesize first-strand cDNA using the PrimeScript^™^ RT Reagent Kit (Takara, Dalian, China). Candidate gene primers for qRT-PCR were designed to span two exons per the reference sequence in BRAD, and the amplified fragment length was limited to approximately 300 bp. The gene *25s* (F: GATTTCTGCCCAGTGCTCTGAA, R: TCTGCCAAGCCCGTTCCCTT) was chosen as the reference gene for the relative quantification of the candidate gene. qRT-PCR was performed in 20-μl reactions that included 10 μl of SYBR^®^ Premix Ex Taq^™^ II (Takara, Dalian, China), 8 μl of template cDNA (12.5 ng/μl), and 1 μl of each primer (10 ng/μl). The PCR conditions were as follows: 95°C for 2 min, followed by 40 cycles of 95°C for 10 s and 60°C for 30 s. A melting curve analysis was also performed. PCR was conducted in a LightCycler^®^ 480 II (Roche). Three individual plants from each line (BC4F2-Y and BC4F2-B) were chosen for quantitative analysis, and the average relative expression levels were calculated. *T* tests were performed to test the significance of differences in expression levels among different samples.

### Candidate gene sequencing and reference sequence analysis

A homologous sequence of the candidate gene was taken from the BRAD database, and the sequence of an orthologous gene was also downloaded from the TAIR database. Primers to amplify the coding region of the candidate gene (including from cDNA and gDNA templates) were designed using the homologous gene sequence taken from BRAD. Mixtures of cDNA and gDNA from each parent and from each BC4F2 offspring group were used as templates for the amplification of the target gene. To amplify gDNA, 0.5 μg of total genomic DNA was used as a template in a standard 50-μl reaction. To amplify cDNA, the template was substituted with 0.3 μg of cDNA. PCR conditions were as follows: pre-denaturation at 94°C for 4 min, followed by 34 cycles of amplification [94°C for 30 s, 55°C for 30 s and 72°C for 2 min] and a final extension at 72°C for 10 min. PCR products were visualized on 1% agarose gels. Target bands were recovered using a gel extraction kit (Sangon Biotech, Shanghai, China), and purified PCR products were incorporated into the pMD18-T vector (Takara). Recombinant vectors were transferred into competent DH5α cells using cold-shock treatment. After blue-white colony screening on solid LB medium, positive clones were chosen and propagated in LB liquid culture before being subjected to PCR amplification. Five positive bacterial solutions for each gene fragment were fully sequenced by Sangon Biotech. The amino acid sequence of the candidate gene was imputed from the coding sequence using the software Primer Premier 5. Sequence comparisons were performed using ClustalW (available at http://www.ebi.ac.uk/clustalw).

## Results

### Whole-genome resequencing analysis

**Fig 1:**
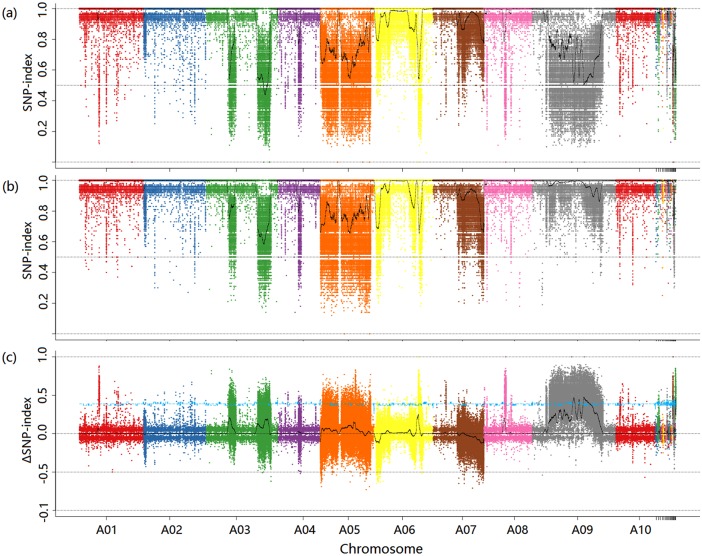
SNP Index and ΔSNP Index Manhattan Plots. (a) SNP index Manhattan plot of the brown-seeded pool from the BC4 population; (b) SNP index Manhattan plot of the yellow-seeded pool from the BC4 population; (c) ΔSNP index Manhattan plot. The blue line indicates the threshold value.

**Fig 2:**
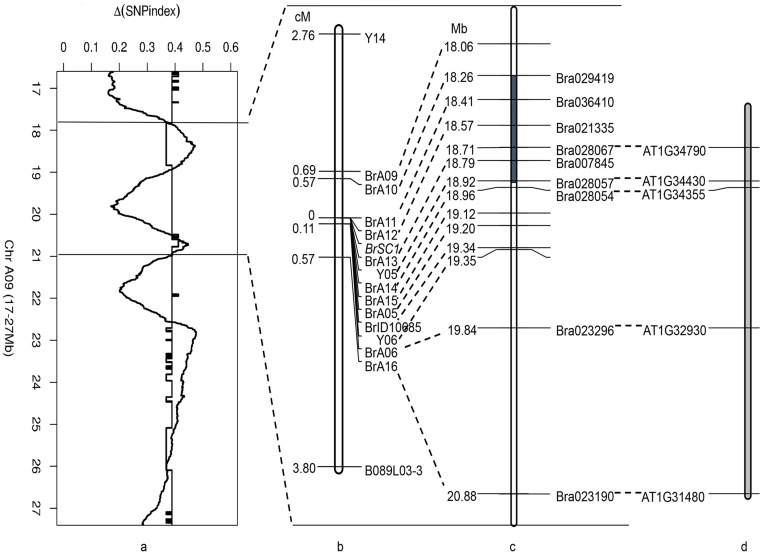
Combination of ΔSNP Index and Genetic Linkage Map for *Brsc1* on A9 of *B*. *rapa*. (a.) Details of the ΔSNP index on chromosome A9. Dentate lines indicate the threshold value; the two parallel black lines delineate the interval from 17.8 to 21.0 Mb on chromosome A9. (b.) Genetic linkage map of linkage markers around *Brsc1* on chromosome A9. The figures on the left indicate the corresponding genetic distances (cM) between markers and *Brsc1*. Some markers are from previous studies (Y05 and Y06: [6]; Y14 and B089L03-3: [23]; BrA05, BrA06 and BrA09: [22]. (c.) Physical map of linkage markers for *Brsc1* and *BrTT1* (*Bra028067*). Figures on the left represent the physical locations (Mb) of linkage markers on chromosome A9. The shaded region indicates the candidate region for *Brsc1*. (d.) Fragment of *A*. *thaliana* chromosome 1. Orthologs of *B*. *rapa* genes are shown on the left, including *TT1* (*AT1G34790*).

After filtering, 30.783 G of clean data was obtained from 31.275 G of raw data. The GC content ranged between 37.26% and 42.45%. The quantity and quality (Q20≥93.97% and Q30≥86.88%, respectively) of the data were sufficient for further analysis. The average depth was >15× for the four samples. After alignment to the reference genome, 1,660,186 SNPs were identified. Based on the 780,061 homozygous SNPs between the two parents, SNP indices for the two BC4 segregant populations’ pools were calculated. A total of 2,429 SNPs were eliminated by applying the following two conditions. First, loci were removed if the SNP indices were <0.3 with an SNP depth <7 in both offspring pools. Second, SNPs were discarded if the SNP indices were missing for either offspring. A ΔSNP index graph was generated from the SNP indices of the two offspring pools (Fig 1). Three candidate intervals (17.83–18.93 Mb; 20.53–20.99 Mb; 22.57–26.09 Mb) that exceeded the threshold value were identified for *Brsc1* on chromosome A9 (Fig 2a).

### Mapping of Brsc1 using molecular markers

BC4 individuals segregated in a 1:1 (yellow-seeded:brown-seeded) ratio. SSR assays were combined with BSA to examine molecular markers that were linked to the *Brsc1* gene. Primers distinguishing brown-seeded DNA bulks and yellow-seeded DNA bulks were believed to be potentially linked to *Brsc1*. Ten SSR primers that revealed polymorphisms between yellow- and brown-seeded bulks were used to amplify gDNA from 12 yellow-seeded plants and 12 brown-seeded plants. This screening eventually identified 8 polymorphic markers, including 7 newly developed markers (designated BrA10-BrA16) and 1 previously available SSR marker (BrID10685) from the BRAD database (Table 1). To more precisely map *Brsc1*, 2 previously identified PCR-based markers (Y14 and B089L03-3) [23] were used to detect recombinants in the BC4 population, which consisted of 1,739 plants. A total of 48 recombinants between Y14 and *Brsc1* and 54 recombinants between B089L03-3 and *Brsc1* were identified. All of the recombinants were then subjected to genotyping with the eight newly developed SSR markers to evaluate their genetic distance from *Brsc1*. BrA11-BrA15 cosegregated with *Brsc1*, and BrA10 and BrID10685, the two closest flanking markers, were 0.57 and 0.11 cM from the target gene, respectively. A genetic linkage map surrounding the *Brsc1* gene was then constructed (Fig 2b). Three SSR markers (BrA05, BrA06, BrA09) [22] and two AFLP (Amplified Fragment Length Polymorphism) markers (Y05, Y06) [6] from previous studies were also incorporated. According to a homologous comparison, *Brsc1* was located between BrA10 (A09:18,255,987–18,255,838) and BrID10685 (A09:19,342,792–19,342,739) (Fig 2c), representing a shorter target interval than previously defined.

**Table 1:** Sequences and Locations of SSR Markers Linked to Brsc1.

SSR Markers	Primer-F(5'-3')	Primer-R(5'-3')	Linkage group (position) in *B*. *rapa*(version 1.1)	size (bp)	Homologous *B*.*rapa* gene and E value	Genetic distance
BrA10	CGTCTTGTACGTCGTGACCT	CTTCTTCCTCTTCCTCGCAC	A09(18,255,987–18,255,838) 274,2e-72,147/150 (98%)	188	Bra029419,208,1e-53,114/117 (97%)	0.575
BrA11	GATTGTTCTTCCTCTCGTCG	TCTTCTGAGGCCACTTGACC	A09(18,407,33–18,407,563) 462,e-129, 233/233 (100%)	152	Bra036410,86,2e-16,49/51 (96%)	0
BrA12	TCTGGACAGAACACCTCACC	TCACGATCTCTCTCTCCTGG	A09(18,570,940–18,571,067) 254,1e-66,128/128 (100%)	163	Bra021335,113,5e-25, 66/69 (95%)	0
BrA13	CTAACCGGCTCCTTATATCC	ACCATTGAGTCAACGTCCTG	A09(18,792,444–18,792,152) 573,e-162,292/293 (99%)	293	Bra007845,135,2e-31,77/80 (96%)	0
BrA14	TACGTGGAGGAGGTCTTAGG	CTGCCTGGACTTCATCTGTG	A09(18,962,948–18,963,069) 234,2e-60,121/122 (99%)	238	Bra028054,234,3e-61,121/122 (99%)	0
BrA15	GTGCCGCAGATAGTAGTAGG	AATACGGAGAGAGAGGTCAGG	A09(19,115,029–19,115,151) 244,2e-63,123/123 (100%)	215	-	0
BrID10685	TTACGAAGAAGAAGCAAAGG	GAGTCGAAAATGGCATGTAT	A09(19,342,792–19,342,739) 100,2e-20,53/54 (98%)	101	-	0.115
BrA16	GAGTTAGCTGCTTCAGTACC	CACCAGAATGACAGCAACTC	A09(20881971–20881748)224,e-124,224/224 (100%)	224	Bra023190,202, 9e-52	0.575

### Dissection of the target region of Brsc1

From a linkage map based on SSR and AFLP markers, Brsc1 was located on chromosome A9 from 18.26 to 19.34 Mb; meanwhile, an adjacent interval from 17.83 to 18.93 Mb for Brsc1 was also detected by whole-genome resequencing. Combining the results from fine mapping and whole-genome resequencing, the target region of *Brsc1* was narrowed to a 678-kb interval (18.26–18.93 Mb), which contains 46 predicted genes in the *B*. *rapa* reference genome (S1 Table). Most of the predicted genes were annotated using information from homologous *A*. *thaliana* genes in TAIR. Among the predicted genes, we found that the gene *Bra028067* (*BrTT1*) (Fig 2) was highly similar to *AT1G34790* (*Transparent Testa 1*, *TT1*), which encodes a zinc-finger protein and is involved in flavonoid biosynthesis in *A*. *thaliana*. *TT1* mutants of *A*. *thaliana* have transparent testa (TT) due to a deficiency in pigment deposition in the endothelium of the seed coat. Therefore, we assumed that the gene *Bra028067* (*BrTT1*) was the most important candidate gene for *Brsc1* and chose it for further study.

### Expression pattern analysis of BrTT1

**Fig 3:**
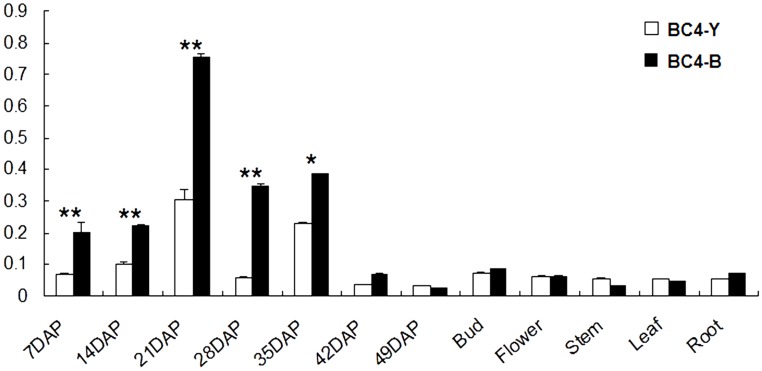
Relative Expression of BrTT1 in Different Organs of BC4F2 Offspring Plants. DAP: days after pollination. The sampling time of different organs (bud, leaf, root, flower, stem) was 7 DAP. **, extremely significant difference between BC4F2-B and BC4F2-Y plants; *, significant difference between BC4F2-B and BC4F2-Y plants.

**Fig 4:**
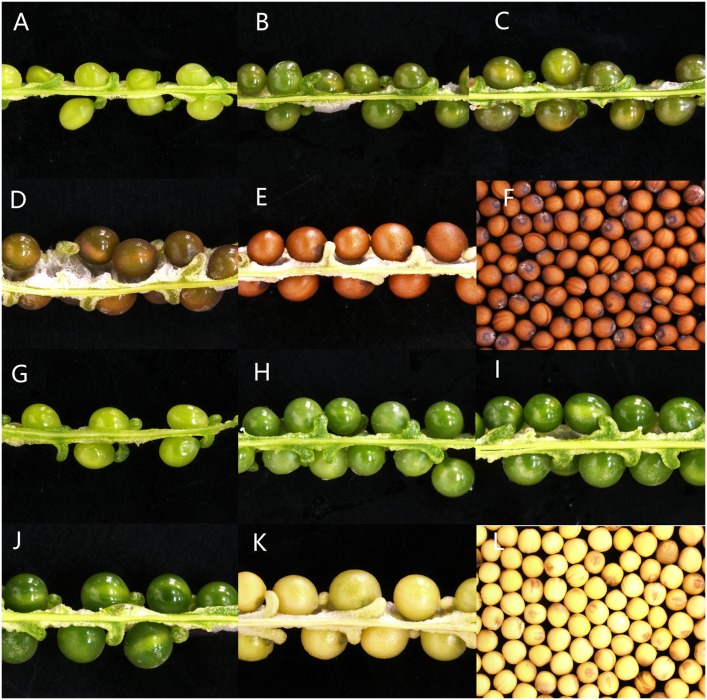
Seeds of Brown- and Yellow-Seeded Plants in BC4F2-B and BC4F2-Y Plants. A, B, C, D, E, F: seeds of BC4F2-B plants; G, H, I, J, K, L: seeds of BC4F2-Y plants; A, G: seeds at 21 DAP; B, H: seeds at 28 DAP; C, I: seeds at 35 DAP; D, J: seeds at 42 DAP; E, K: seeds at 49 DAP; F, L: mature seeds; DAP: days after pollination.

We investigated the expression pattern of *BrTT1* using qRT-PCR in the two offspring groups (BC4F2-B, BC4F2-Y) (Fig 3). A *BrTT1* primer pair for qRT-PCR was designed in Primer Premier 5 using the reference sequence in BRAD (qBrTT1-F: GCTCTTGGCTGAGTCCCTTT; qBrTT1-R: TTGTCACTTACGATGCCGGA). Expression differences in *BrTT1* between BC4F2-B and BC4F2-Y were slight in some organs, such as the flowers, stems, buds, leaves and roots, while those differences in the seeds were notable exceptions. The overall expression in seeds was significantly higher than that in other organs, which indicates that *BrTT1* is specifically expressed in seeds. The expression level of *BrTT1* in BC4F2-B was markedly higher than that in BC4F2-Y during different stages of seed development, especially at 21 DAF and 28 DAF, which represent the early stages before the color staining of the seed coat (Fig 4). The expression level of *BrTT1* peaked at 21 DAF relative to the other stages. A remarkable decline in *BrTT1* expression was detected at 28 DAF, which was more severe in yellow-seeded plants than in brown-seeded plants. The most important stage for the formation of seed coat color may occur at 28 DAF in Dahuang. However, the *BrTT1* expression patterns in yellow-seeded and brown-seeded plants were similar, suggesting that *BrTT1* functions in the same way in the progeny of Dahuang with different seed coat colors. In conclusion, it is reasonable to postulate that *BrTT1* is a potential candidate gene for the seed coat color locus in Dahuang.

### Sequence analysis of BrTT1

**Fig 5:**
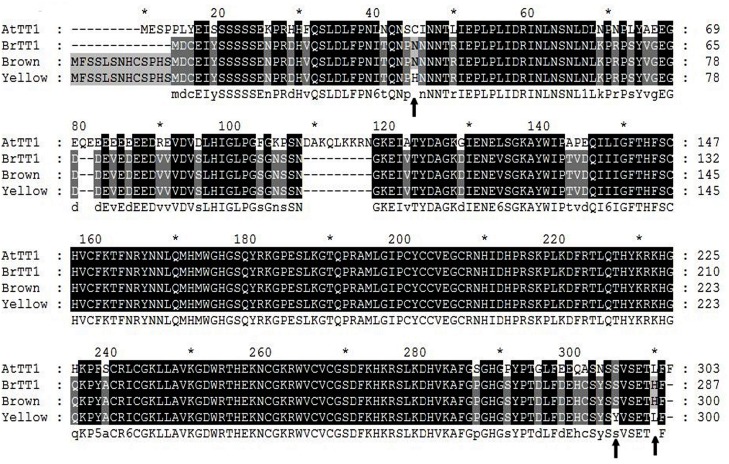
Amino Acid Sequence Alignment Between BrTT1 in B. rapa and AtTT1 in A. thaliana. Arrows indicate single amino acid changes between BC4F2-Y (yellow) and BC4F2-B (brown) plants.

The cDNA and gDNA sequences of the region containing the *BrTT1* coding sequence in the parents and BC4F2 offspring plants were successfully obtained. PCR primers were designed according to the reference sequence in BRAD using Primer Premier 5 (cDNA, F: ATGTTTTCATCACTCTCAAACCACT; R: TTAAAAGAGCGTTTCAGAGACATAAG; gDNA, F: ACTTCAGACATGGGCTCTTTCAAAGATTC; R: AGGGTCGAGAAGGTTACACGTTGCATAT). No sequence differences were found between a given parent and the corresponding BC4F2 offspring with the same seed coat color when subjected to sequence alignment analysis. Reference sequences of *BrTT1* (*Bra028067*) and *AtTT1* (*At1g34790*) from BRAD and TAIR, respectively, were also collected. ClustalW analysis of the gDNA sequences from BC4F2-B and BC4F2-Y plants showed 3 different deletions (of 114 bases, 2 bases and 9 bases) in introns, in addition to single-base changes in the exons of yellow-seeded lines. Two deletions (of 114 bases and 9 bases) were also found in *AtTT1* introns (S1 Fig). Amino acid sequences were predicted from cDNA sequences using Primer Premier 5. Compared with brown-seeded plants, yellow-seeded plants showed three amino acid changes, while most other amino acids were conserved (98.3%). ClustalW showed that 93.67% and 92% of the amino acid sequences of the reference *BrTT1* were identical to sequences of *BrTT1* from BC4F2-B and BC4F2-Y plants, respectively (Fig 5). By comparing the initiation codons of *BrTT1* in Dahuang to those of the reference *BrTT1*, we found two closely linked initiation codons in Dahuang, the second of which was concordant with the reference *BrTT1*. The alternative start codon added an additional 13 amino acids in Dahuang, but whether the added amino acids affect *BrTT1* function in Dahuang remains unknown. Additionally, BLAST alignment showed that the CDS similarity between *BrTT1* and *AtTT1* was as high as 81.71%, while *BrTT1* encodes a protein that shares 81.18% identity with the *AtTT1* protein (Fig 5).

## Discussion

Molecular markers have played an increasingly important role in the fine mapping of target genes over the past few decades. AFLPs and SSRs are two of the most commonly used types of molecular markers [28–30]. To accurately locate target genes, a high-resolution genetic map should be constructed with many tightly linked markers, while a large segregation population is required to determine the genetic distances between markers and the target gene. As more individuals are screened, the representation of the marker location becomes more accurate. In previous mapping results for *Brsc1* in Dahuang, increasing the density of the linkage map for *Brsc1* using SSR markers narrowed the candidate region to chromosome A9. However, we hypothesized that a more precisely defined region was possible. The rapid development of NGS techniques set the stage for whole-genome sequencing. High-throughput sequence data and reduced costs have enabled genotyping by sequencing (GBS), which is a faster and more effective method for detecting target regions with a large number of SNPs [31,32]. Combined with the BSA method, partially coincident location results from whole-genome resequencing verified the mapping results from molecular markers while also improving the accuracy of the latter. Three significant regions for *Brsc1* were detected by resequencing, covering a total of 5.1 Mb on chromosome A9. The best way to identify the region most likely containing *Brsc1* among the three intervals was to analyze the information from linked molecular markers or from the annotation of related genes. A combination of GBS and traditional molecular marker technology may generate more accurate location results [19,21]. Our research provided more evidence for BSA with whole genome resequencing being an effective and rapid method to locate the major QTLs or genes for important traits in diploid plants.

The observed diversity of seed coat color in rapeseed is caused by the differential accumulation of pigments in the endothelium [9,33], which is similar to the TT phenotype in *A*. *thaliana* [34,35]. As *A*. *thaliana* is an important model plant, a detailed study of a series of *TT* genes in *A*. *thaliana* has been conducted [36,37]. *TT* genes in *A*. *thaliana* are mainly involved in the flavonoid biosynthetic pathway and may be divided into three categories: structural genes, regulatory genes and transport genes. Defects in different *TT* genes result in different degrees of variation in testa color [36]. Moreover, considering the close phylogenetic relationship between *Arabidopsis* and *Brassica*, orthologs of *TT* genes may play similar roles in *Brassica* species; quantitative analysis of gene expression supports this inference [33,38]. In *A*. *thaliana*, monogenic mutations may cause remarkable deficiencies in pigment deposition. Meanwhile, some yellow-seeded varieties of *B*. *rapa* and *B*. *juncea* may also be ascribed to mutations in a single major gene, such as TT8 [9,39], *TTG1* [16]. In *B*. *napus*, however, some *TT* genes are likely involved in the staining of seed coats, but there is no direct evidence indicating that one dominant *TT* gene controls seed coat color in these species [33,40,41].

In our research, one ortholog of *AtTT1* was found directly in the target region of *Brsc1*. In *A*. *thaliana*, *TT1* encodes a WIP-type zinc finger protein that functions during the construction of the endothelium and the accumulation of proanthocyanidin pigments as a regulatory gene that interacts with the R2R3-MYB protein TT2. A series of *TT1* mutations leads to the TT phenotype [42,43]. We postulate that *BrTT1* is responsible for the lack of pigment in the seed coats of yellow-seeded Dahuang. Further expression profiling and sequence comparison analyses have partially confirmed this hypothesis, but we cannot yet definitively conclude that this gene is responsible for seed coat color until a detailed functional analysis is performed. Furthermore, *TT* genes are generally single-copy in *Arabidopsis*, whereas multiple copies are found in *Brassica* species [44]. Additionally, multiple orthologs of *TT1* were detected in the *B*. *rapa* genome according to BLAST analysis in the BRAD database, which may complicate its function. As such, more in-depth research on *BrTT1* in Dahuang is warranted. Different kinds of yellow-seeded *B*. *rapa* varieties are cultivated and researched throughout the world. Comparative sequencing of *BrTT1* orthologous genes in different yellow-seeded and brown-seeded *B*. *rapa* varieties may help to identify the mutation sites responsible for the expression differentiation in different *B*. *rapa* varieties. Functional complementation analysis is also required to verify *BrTT1* function in Dahuang.

## Supporting Information

S1 Fig The gDNA sequence alignment between *BrTT1* in *B*. *rapa* and *AtTT1* in *A*. *Thaliana*. The gDNA sequence of *AtTT1* and *BrTT1* are downloaded from TAIR and BRAD database separately; "Brown" indicates the gDNA sequence of *BrTT1* in 09A-126 and BC4-B population; "Yellow" indicates the gDNA sequence of *BrTT1* in Dahuang and BC4-Y population.
(PDF)

S1 Table Genes located in the overlapping candidate region for *Brsc1*. (XLS)
