# Supplementary material for: Fine Mapping and Whole-Genome Resequencing Identify the Seed Coat Color Gene in Brassica rapa
Source: PLoS One. 2016 Nov 9;11(11):e0166464. doi: 10.1371/journal.pone.0166464 (PMC5102352; doi:10.1371/journal.pone.0166464)
Supplement: S1 Fig — The gDNA sequence of AtTT1 and BrTT1 are downloaded from TAIR and BRAD database separately; "Brown" indicates the gDNA sequence of BrTT1 in 09A-126 and BC4-B population; "Yellow" indicates the gDNA sequence of BrTT1 in Dahuang and BC4-Y population. (PDF) [file pone.0166464.s001.pdf]

```
                *      20         *      40         *      60         *      80
AtTT1  : ---------------------------ATGGAGTCACCACCACTATACGAGATATCCTCAAGCTCTTCTTCTGAAAAACCTAGACA :  59
BrTT1  : ---------------------------------------ATGGATTGCGAGATCTACTCAAGCTCTTCTTCTGAAAACCCTAGAGA :  47
Brown  : ATGTTTTCATCACTCTCAAACCACTGTTCACCACATTCAATGGATTGCGAGATCTACTCAAGCTCTTCTTCTGAAAACCCTAGAGA :  86
Yellow : ATGTTTTCATCACTCTCAAACCACTGTTCACCACATTCAATGGATTGCGAGATCTACTCAAGCTCTTCTTCTGAAAACCCTAGAGA :  86
         ATGTTTTCATCACTCTCAAACCACTGTTCACCACATTCAATGGATTGCGAGATCTACTCAAGCTCTTCTTCTGAAAACCCTAGAGA

            *      100        *      120        *      140        *      160        *
AtTT1  : CCATTTCCAATCCCTTGATCTCTTCCCTAACCTCAACCAAAACTCTTGTATCAACAATACCCTAATTGAGCCTTTACCGCTTATTG : 145
BrTT1  : CCACGTCCAATCCCTTGATCTCTTTCCTAACATCACTCAAAACCCTAATAACAACAATACCCGAATCGAACCTTTACCGCTTATCG : 133
Brown  : CCACGTCCAATCCCTTGATCTCTTTCCTAACATCACTCAAAACCCTAATAACAACAATACCCGAATCGAACCTTTACCGCTTATCG : 172
Yellow : CCACGTCCAATCCCTTGATCTCTTTCCTAACATCACTCAAAACCCTCATAACAACAATACCCGAATCGAACCTTTACCGCTTATCG : 172
         CCACGTCCAATCCCTTGATCTCTTTCCTAACATCACTCAAAACCCTAATAACAACAATACCCGAATCGAACCTTTACCGCTTATCG

          180        *      200        *      220        *      240        *        2
AtTT1  : ATCGCATAAACTTGAACTCAAACCTAGACCTAAACCCTAATCCCTTGTATGCGGAAGAAGGAGAGCAAGAGGAGGAAGAAGAAGAA : 231
BrTT1  : ATAGGATCAACTTAAACTCAAACCTAAACCTAAAACCTAGGCCATCGTATGTTGGCGAAGGAGAT------GACGAGGTAGAAGAT : 213
Brown  : ATAGGATCAACTTAAACTCAAACCTAAACCTAAAACCTAGGCCATCGTATGTTGGCGAAGGAGAT------GACGAGGTAGAAGAT : 252
Yellow : ATAGGATCAACTTAAACTCAAACCTAAACCTAAAACCTAGGCCATCGTATGTTGGCGAAGGAGAT------GACGAGGTAGAAGAT : 252
         ATAGGATCAACTTAAACTCAAACCTAAACCTAAAACCTAGGCCATCGTATGTTGGCGAAGGAGATCAAGAGGACGAGGTAGAAGAT

         60        *      280        *      300        *      320        *      340
AtTT1  : GAAGAAGACCGTGAAGTGGACGTGGACTTACACATCGGCCTTCCTGGTTTTGGTAAACCAAGCAATGATGCTAAACAGCTGAAGAA : 317
BrTT1  : GAAGAGGACGTTGTTGTGGACGTGAGCTTACACATCGGCCTTCCTGGTTCCGGTAATTCAAGCAATG------------------- : 280
Brown  : GAAGAGGACGTTGTTGTGGACGTGAGCTTACACATCGGCCTTCCTGGTTCCGGTAATTCAAGCAATG------------------- : 319
Yellow : GAAGAGGACGTTGTAGTGGACGTGAGCTTACACATCGGCCTTCCTGGTTCCGGTAATTCAAGCAATG------------------- : 319
         GAAGAGGACGTTGTAGTGGACGTGAGCTTACACATCGGCCTTCCTGGTTCCGGTAATTCAAGCAATGATGCTAAACAGCTGAAGAA
```

```
              *      360        *      380        *      400        *      420        *
AtTT1  : GAGAAATGGGAAGGAGATCGCCACATATGACGCCGGAAAAGGCATCGAGAATGAACTTTCCGGAAAGGCATACTGGATCCCGGCGC : 403
BrTT1  : --------GGAAAGAGATTGTCACTTACGATGCCGGAAAAGACATCGAAAATGAAGTTTCCGGCAAGGCATATTGGATTCCGACGG : 358
Brown  : --------GGAAAGAGATTGTCACTTACGATGCCGGAAAAGACATCGAAAATGAAGTTTCCGGCAAGGCATATTGGATTCCGACGG : 397
Yellow : --------GGAAAGAGATTGTCACTTACGATGCCGGAAAAGACATCGAAAATGAAGTTTCCGGCAAGTCATATTGGATTCCGACGG : 397
         GAGAAATGGGAAAGAGATTGTCACTTACGATGCCGGAAAAGACATCGAAAATGAAGTTTCCGGCAAGGCATATTGGATTCCGACGG

               440        *      460        *      480        *      500        *
AtTT1  : CGGAGCAAATTCTCATAGGGTTCACTCATTTTTCTTGCCATGTATGCTTCAAGACATTCAATCGCTACAACAATCTTCAGGTACGA : 489
BrTT1  : TGGATCAAATTATAATAGGCTTCACTCATTTTTCTTGCCATGTATGCTTCAAGACATTCAATCGCTACAACAATCTTCAGGTACGA : 444
Brown  : TGGATCAAATTATAATAGGCTTCACTCATTTTTCTTGCCATGTATGCTTCAAGACATTCAATCGCTACAACAATCTTCAGGTACGA : 483
Yellow : TGGATCAAATTATAATAGGCTTCACTCATTTTTCTTGCCATGTATGCTTCAAGACATTCAATCGCTACAACAATCTTCAGGTACGA : 483
         TGGATCAAATTATAATAGGCTTCACTCATTTTTCTTGCCATGTATGCTTCAAGACATTCAATCGCTACAACAATCTTCAGGTACGA

           520        *      540        *      560        *      580        *      600
AtTT1  : GTCAATATATCTCATGCGCATTGCTTTTCCAT--GCACAAACATATATAATAAATTCATCT-TATAGAGTTATATCTCCGGATCTA : 572
BrTT1  : ATCATTATACATTATGCTTGTTGTGTGTCCATGTGCATAACCAGATCATATAGATAACCCTATATATAATTTTCTTTTCTTTTTTT : 530
Brown  : ATCATTATACATTATGCTTGTTGTGTGTCCATGTGCATAACCAGATCATATAGATAACCCTATATATAATTTTCTTTTCTTTTTTT : 569
Yellow : ATCATTATATATTATGCTTGTTGTGTGTCCATGTGCACAACCAGATCATATAGATAACCCTATATATAATTTTCTTTTCTTTTTTT : 569
         ATCATTATACATTATGCTTGTTGTGTGTCCATGTGCACAACCAGATCATATAGATAACCCTATATATAATTTTCTTTTCTTTTTTT

                *      620        *      640        *      660        *      680
AtTT1  : ATGTTATGAGTTT-ATTCATATCTATATA-TATACATATA-TATATATATATATATATATA----------------TATATATAT : 639
BrTT1  : TTCTGAGAAATTTTGCTTAGAAATATATAATTTTCATATACTATTTATATCTCCGTATCTAATGTTAGGACTTTGTTCATACATAA : 616
Brown  : TTCTGAGAAATTTTGCTTAGAAATATATAATTTTCATATACTATTTATATCTCCGTATCTAATGTTAGGACTTTGTTCATACATAA : 655
Yellow : T-CTGAGAAATTTTGCTTAGAAATATATAATTTTCATATACTATTTATATCTCCGTATCTAATGTTAGGATTTTGTTCATACATAA : 654
         TTCTGAGAAATTTTGCTTAGAAATATATAATTTTCATATACTATTTATATCTCCGTATCTAATGTTAGGACTTTGTTCATACATAA
```

(To be continued)

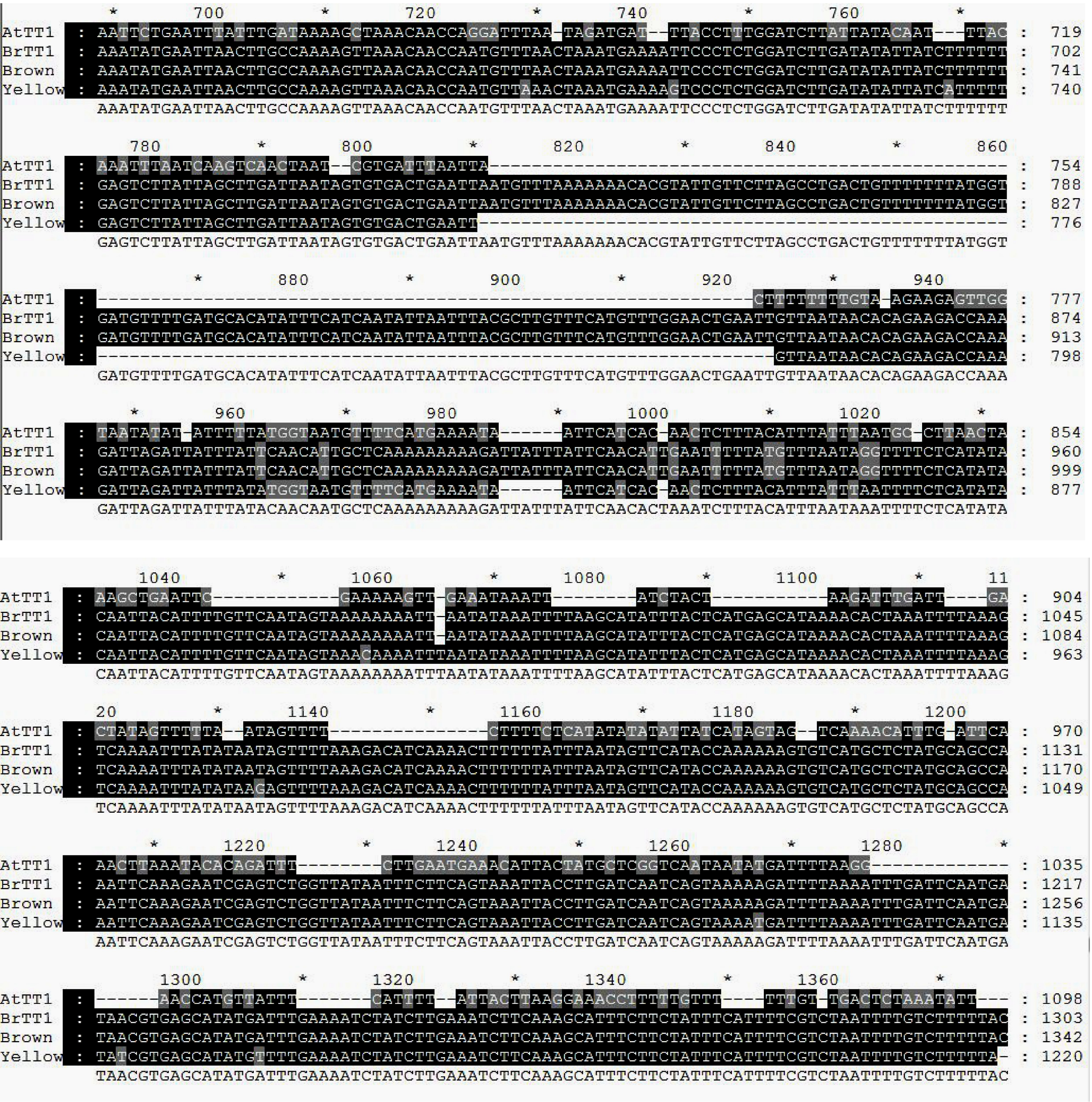

(To be continued)

```
         1380        *     1400          *     1420          *     1440          *     1460
AtTT1  : ------------------------ATGAATATAGATGCACATGTGGGGACATGGTTCACAATACAGGAAAGGACCGGAGTCACT : 1158
BrTT1  : ATATGAAAACCTGGTTGATTCCAAATATGAATGCAGATGCACATGTGGGGCCACGGTTCACAATACAGGAAAGGACCAGAGTCACT : 1389
Brown  : ATATGAAAACCTGGTTGATTCCAAATATGAATGCAGATGCACATGTGGGGCCACGGTTCACAATACAGGAAAGGACCAGAGTCACT : 1428
Yellow : --------ACCTGGTTGATTCCAAATATGAATGCAGATGCACATGTGGGGCCACGGTTCACAATACAGGAAAGGACCAGAGTCACT : 1298
         ATATGAAAACCTGGTTGATTCCAAATATGAATGCAGATGCACATGTGGGGCCACGGTTCACAATACAGGAAAGGACCAGAGTCACT

                *     1480          *     1500          *     1520          *     1540
AtTT1  : GAAAGGCACACAGCCACGAGCCATGTTAGGGATCCCTTGTTACTGCTGCGTTGAAGGGTGCAGGAACCACATTGACCATCCTCGTT : 1244
BrTT1  : GAAAGGGACTCAGCCAAGAGCCATGCTAGGCATCCCTTGTTACTGCTGCGTTGAAGGGTGCAGGAACCACATCGATCATCCTCGGT : 1475
Brown  : GAAAGGGACTCAGCCAAGAGCCATGCTAGGCATCCCTTGTTACTGCTGCGTTGAAGGGTGCAGGAACCACATCGATCATCCTCGGT : 1514
Yellow : GAAAGGGACTCAGCCAAGAGCCATGCTTGGCATCCCTTGTTACTGCTGCGTTGAAGGGTGCAGGAACCACATCGATCATCCTCGGT : 1384
         GAAAGGGACTCAGCCAAGAGCCATGCTAGGCATCCCTTGTTACTGCTGCGTTGAAGGGTGCAGGAACCACATCGATCATCCTCGGT

          *     1560          *     1580          *     1600          *     1620          *
AtTT1  : CCAAGCCACTGAAAGACTTTAGGACGCTCCAAACGCACTACAAACGCAAACACGGACACAAACCCTTCTCGTGTCGCCTTTGCGGT : 1330
BrTT1  : CCAAGCCACTCAAAGACTTCCGAACGCTCCAAACGCACTATAAGCGCAAACACGGCCAAAAGCCTTATGCGTGTCGCATTTGCGGT : 1561
Brown  : CCAAGCCACTCAAAGACTTCCGAACGCTCCAAACGCACTATAAGCGCAAACACGGCCAAAAGCCTTATGCGTGTCGCATTTGCGGT : 1600
Yellow : CCAAGCCACTCAAAGACTTCCGAACGCTCCAAACGCACTATAAGCGCAAACACGGCCAAAAGCCTTATGCGTGTCGCATTTGCGGT : 1470
         CCAAGCCACTCAAAGACTTCCGAACGCTCCAAACGCACTATAAGCGCAAACACGGCCAAAAGCCTTATGCGTGTCGCATTTGCGGT

           1640          *     1660          *     1680          *     1700          *     1720
AtTT1  : AAGCTTTTGGCTGTCAAGGGCGATTGGCGAACACATGAGAAGAATTGTGGAAAACGTTGGGTTTGCGTTTGCGGTTCTGATTTTAA : 1416
BrTT1  : AAGCTCTTGGCAGTTAAGGGAGATTGGCGAACTCACGAGAAGAACTGTGGGAAACGTTGGGTTTGTGTTTGCGGTTCCGATTTTAA : 1647
Brown  : AAGCTCTTGGCAGTTAAGGGAGATTGGCGAACTCACGAGAAGAACTGTGGGAAACGTTGGGTTTGTGTTTGCGGTTCCGATTTTAA : 1686
Yellow : AAGCTCTTGGCAGTTAAGGGAGATTGGCGAACTCACGAGAAGAACTGTGGGAAACGTTGGGTTTGTGTTTGCGGTTCCGATTTTAA : 1556
         AAGCTCTTGGCAGTTAAGGGAGATTGGCGAACTCACGAGAAGAACTGTGGGAAACGTTGGGTTTGTGTTTGCGGTTCCGATTTTAA

                 *     1740          *     1760          *     1780          *     1800
AtTT1  : ACACAAACGTTCTCTTAAGGACCATGTTAAGGCGTTTGGGTCTGGTCATGGGCCTTATCCAACTGGTTTGTTTGAAGAGCAGGCTT : 1502
BrTT1  : ACACAAACGCTCCCTTAAAGACCATGTTAAGGCTTTTGGACCTGGTCATGGGTCTTATCCGACCGATTTGTTTGATGAGCACTGCT : 1733
Brown  : ACACAAACGCTCCCTTAAAGACCATGTTAAGGCTTTTGGACCTGGTCATGGGTCTTATCCGACCGATTTGTTTGATGAGCACTGCT : 1772
Yellow : ACACAAACGCTCCCTTAAAGACCATATTAAGGCTTTTGGACCTGGTCATGGGTCTTATCCGACCGATTTGTTTGATGAGCACTGCT : 1642
         ACACAAACGCTCCCTTAAAGACCATGTTAAGGCTTTTGGACCTGGTCATGGGTCTTATCCGACCGATTTGTTTGATGAGCACTGCT

            *     1820          *     1840
AtTT1  : CTAATTCATCTGTCTCCGAGACTTTGTTTTTTTAA : 1537
BrTT1  : CATACTCTTCTGTCTCTGAAAC---GCACTTTTAA : 1765
Brown  : CATACTCTTCTGTCTCTGAAAC---GCACTTTTAA : 1804
Yellow : CATACTCTTATGTCTCTGAAAC---GCTCTTTTAA : 1674
         CATACTCTTCTGTCTCTGAAACTTTGCACTTTTAA
```

(End)

Supplementary Fig. 1 The gDNA sequence alignment between *BrTT1* in different materials of *B. rapa* and *AtTT1* in *A. Thaliana*.

The gDNA Sequences of *AtTT1* and *BrTT1* are downloaded from TAIR and BRAD database separately; "Brown" indicates the gDNA sequence of *BrTT1* in 09A-126 and BC4-B population; "Yellow" indicates the gDNA sequence of *BrTT1* in Dahuang and BC4-Y population.
